# Visual cortex responses reflect temporal structure of continuous quasi-rhythmic sensory stimulation

**DOI:** 10.1016/j.neuroimage.2016.11.043

**Published:** 2017-02-01

**Authors:** Christian Keitel, Gregor Thut, Joachim Gross

**Affiliations:** Centre for Cognitive Neuroimaging, Institute of Neuroscience and Psychology, University of Glasgow, 58 Hillhead Street, G12 8QB Glasgow, UK

**Keywords:** Brain oscillation, Entrainment, Non-invasive brain stimulation (NIBS), Steady-state response (SSR), Frequency tagging, Brain-computer interface

## Abstract

Neural processing of dynamic continuous visual input, and cognitive influences thereon, are frequently studied in paradigms employing strictly rhythmic stimulation. However, the temporal structure of natural stimuli is hardly ever fully rhythmic but possesses certain spectral bandwidths (e.g. lip movements in speech, gestures). Examining periodic brain responses elicited by strictly rhythmic stimulation might thus represent ideal, yet isolated cases. Here, we tested how the visual system reflects quasi-rhythmic stimulation with frequencies continuously varying within ranges of classical theta (4–7 Hz), alpha (8–13 Hz) and beta bands (14–20 Hz) using EEG. Our findings substantiate a systematic and sustained neural phase-locking to stimulation in all three frequency ranges. Further, we found that allocation of spatial attention enhances EEG-stimulus locking to theta- and alpha-band stimulation. Our results bridge recent findings regarding phase locking (“entrainment”) to quasi-rhythmic visual input and “frequency-tagging” experiments employing strictly rhythmic stimulation. We propose that sustained EEG-stimulus locking can be considered as a continuous neural signature of processing dynamic sensory input in early visual cortices. Accordingly, EEG-stimulus locking serves to trace the temporal evolution of rhythmic as well as quasi-rhythmic visual input and is subject to attentional bias.

## Highlights

• Dynamic visual stimuli constitute large parts of our perceptual experience.
• Strictly rhythmic dynamics condense in EEG-recorded mass-neural activity.
• We tested how stimuli with fluctuating rhythms reflect in the EEG.
• We found that the EEG allows tracing two quasi-rhythmic stimuli in parallel.
• Dynamics of attended stimuli may be tracked with greater temporal precision.

## 1 Introduction

The Human visual system excels in organising the massive and continuous inflow of sensory impressions into meaningful and behaviourally relevant entities. Its capability of exploiting the rich temporal structure of dynamic visual input supports this effort extensively (Blake and Lee, 2005, Buracas et al., 1998, Mazzoni et al., 2011). Temporal structure aides in separating figure from ground (Alais et al., 1998, Guttman et al., 2007), extrapolating the origin and destination of moving objects (Nijhawan, 1994, Whitney, 2002) and increasing sensitivity to upcoming sensory input (Correa and Nobre, 2008, Lasley and Cohn, 1981). Despite these vital aspects of visual perception, little is known about how neural processing of continuous visual stimulation unfolds in time.

Classically, neuroimaging studies have focussed on neural responses to visual transients owing to the fact that these allow for better experimental control (Rust and Movshon, 2005). Current day Human visual neuroscience nevertheless features two lines of research on dynamic visual input processing: Entrainment studies focus on the ability of the visual system to synchronize intrinsic rhythms of the brain, such as theta (4–7 Hz) or alpha (8–13 Hz), to temporal regularities embedded in continuous visual input (Adrian and Matthews, 1934, Notbohm et al., 2016, Thut et al., 2011). Research into auditory processing contributes that this brain-stimulus coupling affords efficient coding due to the deterministic nature of the stimulus (Henry and Herrmann, 2014, Schroeder and Lakatos, 2009). Further, it enables precise predictions of future stimulus occurrences when using visual presentation rates within theta (Cravo et al., 2013) or alpha bands (Spaak et al., 2014).

Another line of research, using the frequency-tagging approach, probes influences of changes in emotional (Bekhtereva and Muller, 2015, Keil et al., 2003, Keil et al., 2009a) or cognitive states, e.g. attention (Keitel et al., 2013a, Kim et al., 2007, Stormer et al., 2014), perceptual qualities (Jacoby et al., 2012, Parks et al., 2011, Porcu et al., 2014) or stimulus properties, e.g. face orientation (Rossion et al., 2012), on early visual processing by means of continuous stimulus-driven brain activity, termed steady-state responses (*SSRs*; reviewed in Norcia et al., 2015; Regan, 1966).

Both lines of research have in common that experiments typically feature strictly rhythmic visual stimulation. However, this type of stimulation may only represent an ideal case that is predominantly met by artificially generated sensory input (Blake and Lee, 2005). In contrast, natural sensory input likely exhibits quasi-periodic temporal structure at best, meaning that its spectral composition can vary over time (Butts et al., 2007, Kayser et al., 2003, Mazzoni et al., 2011).

Prominent examples are the visual components of audio-visual speech (Chandrasekaran et al., 2009). Dynamic speech-related stimuli such as gestures (Biau and Soto-Faraco, 2015) and quasi-periodic lip movements (Zion Golumbic et al., 2013) aid in comprehending speech under challenging listening conditions. Frequency variations, inherent to these stimuli, convey information and thus are functionally relevant (Giraud and Poeppel, 2012). For example, human observers are able to discriminate different languages (Soto-Faraco et al., 2007) or even perform speech-reading (Summerfield, 1992) using only visual information.

**Fig. 1:**
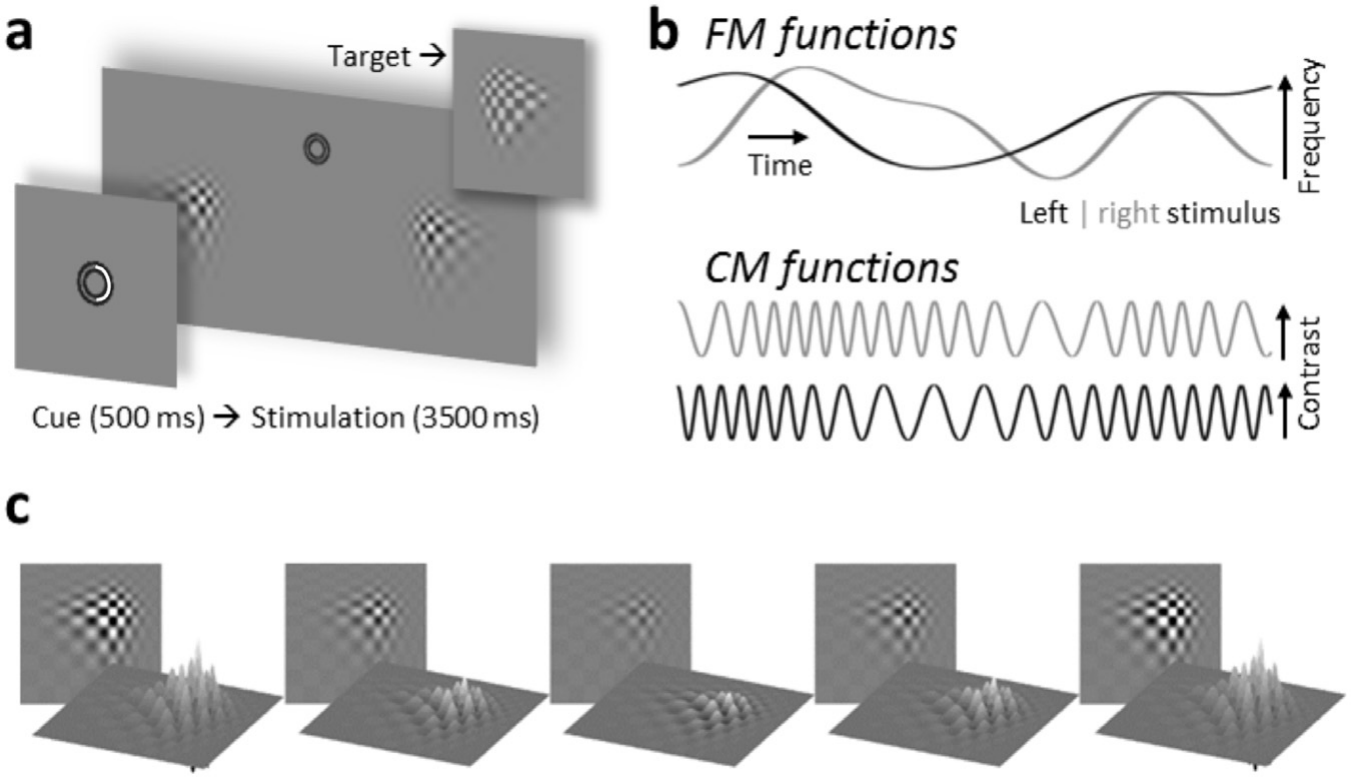
Details of experimental stimulation (a) Trial time course. Central cue presentation (white arc=*attend right*; arc cue colour was green in original stimulation) precedes continuous streams of contrast modulating patches. Upper right inset gives an example of target (and distracter) appearances. (b) Time series depict random band-limited frequency fluctuations (FM functions, top graphs) in periodic contrast modulation functions (CMFs, bottom graphs) for left and right stimuli on a given trial. (c) Left stimulus traversing one CMF peak-to-peak cycle.

Despite the functional relevance of frequency variations, how the Human visual system processes dynamic quasi-rhythmic input has so far attracted little attention (but see Chang et al., 2015; Goncalves et al., 2014; Schall et al., 2009). In the present study, we aimed to systematically characterise neural activity that indicated processing of visual stimuli exhibiting quasi-rhythmic contrast changes within classical theta (4–7 Hz), alpha (8–13 Hz) or beta bands (14–20 Hz). On each trial of the experiment we presented two frequency-varying stimuli, one in each lower visual hemifield, for several seconds while participants were randomly cued to attend to either the left or right stimulus only to perform a visual detection task (see Fig. 1). An additional condition, in which stimuli changed contrast rhythmically at 10 Hz (left) and 12 Hz (right stimulus) served to qualify putative attentional biases of neural responses to quasi-rhythmic stimulation against known gain modulations of SSRs.

In line with a recent study on cortical entrainment to visual elements of speech (Park et al., 2016) we anticipated full-scalp electroencephalographic (EEG) recordings to reflect theta-band stimulation. Because periodic brain responses can be driven over a wide range of frequencies up to 100 Hz using strictly rhythmic stimulation (Herrmann, 2001), we assumed similar responses to stimulation above the theta range. Also, experiments using speech-related visual stimulation have so far only indirectly inferred effects of attention on corresponding neural responses (Crosse et al., 2015, Park et al., 2016). Our paradigm allowed for directly assessing influences of visuo-spatial attention on brain responses to concurrent quasi-rhythmic stimuli within frequency ranges relevant for visual speech perception and beyond.

In brief, we pursued the following specific objectives:

1. Replicate attentional modulations of two well-established SSR measures (Kashiwase et al., 2012, Kim et al., 2007, Porcu et al., 2013), spectral power and inter-trial phase consistency (ITC), in data from our strictly-rhythmic stimulation conditions.
2. Quantify SSRs from strictly-rhythmic stimulation conditions by evaluating neural phase-locking to stimulation (termed *EEG-stimulus locking*) expressed as spectral cross-coherence (XCOH). Compare attention effects on EEG-stimulus locking with effects obtained from (1).
3. Quantify a similar measure (based on XCOH) of EEG-stimulus locking to visual stimulation with varying frequencies (theta, alpha and beta) and test for differences between frequency bands.
4. Examine gain effects on EEG-stimulus locking when participants attended vs ignored corresponding frequency-varying stimuli. Compare gain effects between brain responses driven by rhythmic and quasi-rhythmic stimulation.

**Fig. 2:**
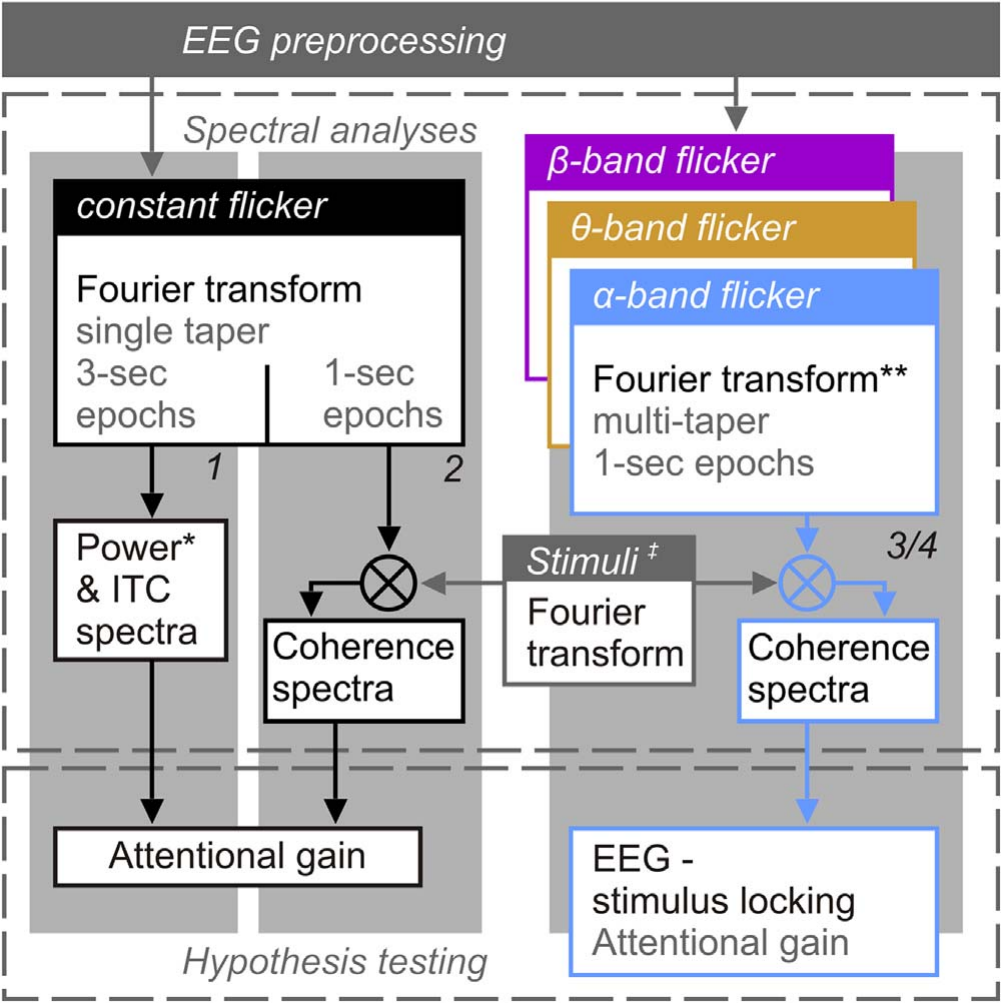
Analyses flow chart. *Power spectra were computed from averaged epochs. ITC=inter-trial coherence. **Prior to Fourier transforms EEG time series were resampled to 100 Hz to match the stimulus sampling rate. ^ǂ^ “Stimuli” refers to the two contrast modulation functions (CMFs) per trial. Fourier transforms of CMFs used the same parameters as the corresponding EEG data (1-s epochs windowed using single Hanning tapers for rhythmic-stimulation conditions vs multi-tapers for quasi-rhythmic stimulation). Numbered grey backdrops (*1*, *2* and *3/4*) illustrate which flow corresponds to which of the four analyses steps.

Underlying data analyses are described in detail in Section 2.6 and illustrated in Fig. 2.

## 2 Methods

### 2.1 Participants

Twenty-two volunteers with normal or corrected-to-normal vision and no history of neurological diseases or injury participated in the study. After pre-processing of EEG and behavioural data we excluded 4 participants due to excessive eye movements during recordings and one participant due to chance level performance in the visual detection task. Data of the remaining participants (N=17, 13 women; median age=22 yrs, range=19–32 yrs) entered further analyses.

The ethics committee of the College of Science & Engineering at the University of Glasgow approved of all procedures (application no. 300140020). Participants gave informed written consent prior to the experiment.

### 2.2 Stimulation

Stimuli were presented on a 21-inch cathode ray tube screen, using a refresh rate of 100 frames per second and a resolution of 1024×768 pixel (width×height). Experimental stimulation comprised two peripherally presented “blurry” checkerboard-like patches (horizontal/vertical diameter=6°/4.4° of visual angle) as well as two small concentric circles (maximum eccentricity=0.4°; luminance<1 cd/m^2^) that served as a central fixation point. Patches were positioned in lower left and right visual quadrants at a centre-to-centre eccentricity of 4.4° relative to fixation (Fig. 1a). All stimuli were presented against a grey background (luminance=6.5 cd/m^2^). Patches were generated by MATLAB’s (The Mathworks, Natick, MA, USA) wavefun2 function yielding 2-dimensional 10-th order Daubechies wavelets computed in 4 iterations. We opted for this type of stimuli because their texture provided an optimal basis for a contrast modulation as described below. In addition, their characteristic “distortion” allowed positioning the patches in such a way that their spatial frequency decreased as a function of eccentricity from fixation, thus, approximating the gradient of sparser visual resolution towards para-foveal regions.

Both patches underwent periodic contrast changes in the course of each trial: Contrast, i.e. stimulus visibility, varied between a maximum of 90% Michelson contrast (peak luminance=29.1 cd/m^2^) and a minimum of 10% (peak luminance=7.5 cd/m^2^). As illustrated in Fig. 1b Patch contrast changed incrementally on each frame of the presentation to approximate sinusoidal contrast modulations (Andersen and Muller, 2015). Crucially, the mean rate of change differed between experimental conditions: In a control condition that served to compare results with earlier frequency-tagging studies (Keitel et al., 2013a, Kim et al., 2007, Müller et al., 1998) contrast modulation occurred with a constant frequency of 10 Hz for the left and 12 Hz for the right stimulus (“constant flicker” condition). In the remaining conditions we tested quasi-rhythmic stimulation limited to theta- (4–7 Hz), alpha- (8–13 Hz) and beta-bands (14–20 Hz). To this end, both patches changed contrast with frequencies that varied along pre-defined random trajectories (=‘frequency modulation functions’ or ‘FMFs’). Each trial featured two pre-defined FMFs (see Fig. 1b) that were up-sampled to match the screen refresh rate from two separate random processes sampled at 2 Hz. Random processes were generated for each trial anew such that one set of FMFs only occurred once for each participant.

In FMFs the maximum velocity of frequency changes was limited to 3.2 Hz/s (theta), 5.1 Hz/s (alpha) and 6.0 Hz/s (beta), i.e. roughly corresponding to a full crossing of respective bandwidths per second. The correlation between the two FMFs on any given trial was kept within the range of ±0.05 (Pearson correlation coefficient *r*) because, in quasi-rhythmic conditions, frequencies of both patches varied within the same frequency band. Thus constraining covariation of frequency changes in patch contrast modulation effectively controlled for prolonged periods of patches flickering at similar frequencies or having similar trajectories.

Finally, FMFs were applied to sinusoidal carriers yielding contrast modulation functions (CMFs, Fig. 1b) sampled at the screen refresh rate. This allowed for a presentation similar to the constant flicker condition with smooth frame-wise changes in stimulus contrast (Fig. 1c). As a consequence of the uniqueness of FMFs, CMFs were generated separately for each trial and were never repeated.

### 2.3 Procedure and task

Participants were seated comfortably in an acoustically dampened and electromagnetically shielded chamber and directed gaze towards the fixation ring on the computer screen. At the beginning of each trial, a green semi-circle (RGB: 0, 230, 0) appeared between fixation circles for 0.5 s cueing participants to attend to the left or right stimulus (Fig. 1a). Subsequently, the two flickering patches were presented for 3.5 s. After flicker offset, the fixation ring remained on screen for an extra 0.7 s allowing participants to blink before the next trial started.

Participants were instructed to respond to occasional brief “flashes” of the cued stimulus (=targets) while ignoring similar events in the other stimulus (=distracters). For that purpose, local stimulus luminance was capped at ±10% of background luminance and affected areas were replaced with uniform grey tones (luminance=±30% of background) for a 0.3 s interval (Fig. 1a *inset*). Targets and distracters occurred in one third of all trials and up to 2 times in one trial with a minimum interval of 0.8 s between subsequent onsets. Responses were recorded as space-bar presses on a standard keyboard. Participants started responding with either their left or right hand. Halfway through the experiment they were then instructed to respond with the other hand.

In our experiment we manipulated the two factors *attended position* (left vs. right patch) and *stimulation frequency* (constant, theta, alpha and beta) in a fully balanced design. Trials of the resulting eight conditions were presented in a pseudo-randomized order. In total we presented 576 trials (=72 trials per condition) divided into 8 blocks (~6 min each). Prior to the experiment, participants performed a training of at least one block. They received feedback regarding average hit rate and reaction time after each training and each experimental block.

### 2.4 Behavioural data recording and analyses

Responses were considered a ‘hit’ when a button press occurred between 0.2 and 1 s after target onset. We further defined correct rejections as omitted responses to distracter stimuli. Based on these data, we calculated the response accuracy (ACC) as the ratio of correct responses (number of hits and correct rejections) to the total number of targets and distracters for each condition and participant according to:

(1) $$\mathrm{ACC}=\;\frac{N_{\mathrm{Hits}}+N_{\mathrm{Correct}\;\mathrm{Rejections}}}{N_{\mathrm{Targets}}+N_{\mathrm{Distracters}}}$$

Accuracies were subjected to a two-way repeated measures analysis of variances (ANOVA) with factors of *attended position* (left vs. right) and *stimulation frequency* (constant, theta, alpha, or beta). Reaction times (RTs) were analysed accordingly. Note that RT analyses were based on median RTs per participant and condition to account for the typical left skew of RT distributions.

In all repeated measures ANOVAs conducted in this study, the Greenhouse–Geisser (GG) adjustment of degrees of freedom was applied to control for violations of sphericity (Greenhouse and Geisser, 1959). Original degrees of freedom, corrected p-values (p_GG_) and the correction coefficient epsilon (ε_GG_) are reported.

### 2.5 Electrophysiological data recording and preprocessing

EEG was recorded from 128 scalp electrodes that were mounted in an elastic cap using a BioSemi ActiveTwo system (BioSemi, Amsterdam, Netherlands) set to a sampling rate of 512 Hz. Lateral eye movements were monitored with a bipolar outer canthus montage (horizontal electro-oculogram). Vertical eye movements and blinks were monitored with a bipolar montage positioned below and above the right eye (vertical electro-oculogram). From continuous data, we extracted epochs of 5 s starting 1 s before patch onset. In further pre-processing, we excluded trials containing transient targets and distracters (24 per condition) as well as epochs with horizontal and vertical eye movements exceeding 20 μV (~2° of visual angle) or containing blinks.

We further applied the ‘fully automated statistical thresholding for EEG artefact rejection’ (FASTER, Nolan et al., 2010). This procedure corrected or discarded epochs with residual artefacts based on statistical parameters of the data. Artefact correction employed a spherical-spline-based channel interpolation. In addition to the earlier criteria, epochs with more than 12 artefact-contaminated electrodes were also excluded from analysis. For each participant FASTER interpolated up to 5 globally contaminated electrodes (median=2) and an average of up to 5.4 intermittently contaminated electrodes (median=3.4) per epoch.

In summary, from 48 available epochs per condition we discarded a median of 11.5 (24%) per participant with a between-subject range of 5.3 to 20.6 epochs (11–43%). Note that high rates of trial rejections can be expected in covert attention studies that include a thorough control of eye movements (see e.g. Keitel et al., 2013a). We refrained from artificially equating epoch numbers across conditions because within-participant variation with a median range of ±4 trials (±11%) around individual means remained small. Finally, data were re-referenced to average reference. Basic data processing steps such as extraction of epochs from continuous recordings, re-referencing and plotting of scalp iso-contour voltage maps made use of EEGLAB (Delorme and Makeig, 2004) in combination with custom routines written in MATLAB.

### 2.6 Electrophysiological data analyses

#### 2.6.1 Common analyses procedures

EEG data analyses were carried out in Fieldtrip (Oostenveld et al., 2011). All analyses steps are illustrated in Fig. 2. From pre-processed artefact-free epochs (5 s) we extracted segments of 3 s starting 0.5 s after patch onset. Data prior to stimulation (1 s) were omitted because they only served to identify eye movements shortly before and during cue presentation. The initial 0.5 s of stimulation were excluded to attenuate the influence of stimulus-onset evoked activity on EEG spectral decomposition. We further disregarded the final 0.5 s of original epochs because stimulation ceased after 3.5 s. In principle, this final period would have afforded investigating offset responses or a post-offset stimulus-induced reverberation (Spaak et al., 2014). However, participants frequently blinked their eyes shortly after stimulation offset as instructed (see Section 2.3), thus, disallowing further EEG analyses.

Re-epoched 3-s data segments were converted to scalp current densities, a reference-free measure, that typically yields topographies with more circumscribed maxima (Ferree, 2006, Kayser and Tenke, 2015) as has been demonstrated also for continuous periodic brain responses (Keitel et al., 2013b). Scalp current density computations involved the Fieldtrip function *ft_scalpcurrentdensity* using the ‘spline’ method (Perrin et al., 1987) while setting the lambda parameter to 10^−4^.

Statistical significance of attention effects and stimulus-locking (see box *Hypothesis testing* in Fig. 2) was assessed by means of cluster-based permutation tests (Maris and Oostenveld, 2007) using *N*=1000 random permutations. Dependent upon specific contrasts data were either clustered across <channel×frequency>doublets (EEG-stimulus locking – XCOH) or single values per channel (power, ITC and XCOH in case of SSR attention effects; XCOH in case of EEG-stimulus locking attention effects) using an average neighbourhood of 7.3 channels. Resulting probabilities were corrected for two-tailed testing.

For a second-level comparison of attention effects across measures (power, ITC and XCOH) or conditions (constant vs. frequency-varying stimulation) we computed attention modulation indices (AMIs) for each measure. Prior to aggregation, measures were collapsed across electrodes as derived from above described cluster analyses. Specifically, attentional gain was quantified as:

(2) $${\mathrm{AMI}}_{\mathrm{ijk}}=\frac{X_{\mathrm{ijk}}^{\mathrm{att}}-\;X_{\mathrm{ijk}}^{\mathrm{unatt}}}{X_{\mathrm{ijk}}^{\mathrm{att}}+\;X_{\mathrm{ijk}}^{\mathrm{unatt}}}$$

where *X* represents the numerical value of a given measure for each stimulation condition *i* and participant *j* under conditions in which the corresponding stimulus *k* was attended (superscript *att*) or unattended (*unatt*). The resulting AMI has previously been used to evaluate gain effects (Kastner et al., 2001, Keitel et al., 2013b). It effectively normalizes inter-individual variance within and between measures and thus serves to retain the net attention effect. AMIs were subjected to repeated-measures analyses of variances (ANOVAs). Specific factorial designs are reported below. Dependencies between measures were further investigated by fitting robust linear models to the data using the MATLAB built-in function *fitlm* (enabling *RobustOpts*, otherwise using defaults). In all regression analyses reported here, outliers were excluded by evaluating Cook’s distance measure (Cook, 1977). In case of exclusions, we provide the number of outliers and report statistics based on outlier-removed data. Outlier points are further indicated in scatter plots illustrating regressions.

#### 2.6.2 Step 1: rhythmic-stimulation driven SSRs – power and inter-trial phase coherence (ITC)

First, we focused our analyses on those two experimental conditions (*attend left* vs *attend right*) that featured stimulus contrast modulations at constant rates of 10 Hz (left stimulus) and 12 Hz (right stimulus). Following a typical approach of SSR analyses in spatial attention paradigms, detrended (i.e. linear trend removed) data segments were averaged for each subject, both conditions and each EEG channel separately. Fourier transforms of untapered averaged time series yielded spectral power estimates, i.e. squared absolute values of complex Fourier spectra (Gross, 2014), with a frequency resolution of 1/3 Hz. For 10 and 12-Hz components separately, cluster-based permutation tests identified electrodes that showed systematic gain effects when contrasting attended vs unattended conditions. SSR amplitudes (square-root of power) at these electrode clusters served to calculate AMIs (see Eq. (2)).

Additionally, we determined AMIs based on electrode clusters showing substantial modulations of ITC by attention (Delorme and Makeig, 2004). To this end, detrended 3-s EEG scalp current density time series were subjected to Fourier transforms prior to averaging across trials. The absolute value of the resulting complex quantity expressed the inter-trial phase coherence at each frequency across trials according to:

(3) $$\mathrm{ITC}\left(f\right)=\;\left|\frac{1}{N}\sum_{n=1}^{N}\frac{C_{n}\left(f\right)}{\left|C_{n}\left(f\right)\right|}\right|$$

where C_*n*_*(f)* is the complex Fourier coefficient of trial *n* of *N* at frequency *f* and |.| indicates the absolute value (Gross, 2014). Phase locking as a measure of SSR modulation has been introduced to SSR analyses more recently (Kim et al., 2007, Nozaradan et al., 2012). Both, SSR power and phase locking have demonstrated sensitivities to top-down influences on sensory processing (Kashiwase et al., 2012, Porcu et al., 2013). Similar to SSR amplitude or power, phase locking values can be visualized as spectra that typically display narrow peaks at stimulation frequencies (Nozaradan et al., 2012, Ruhnau et al., 2016).

#### 2.6.3 Step 2: rhythmic-stimulation driven SSRs – EEG-stimulus cross-coherence (XCOH)

In addition to the two established SSR measures described above we quantified SSRs by taking into account the stimuli themselves: The rhythmic variations in stimulus contrast were described precisely by a sinusoidal function of frequency 10 or 12 Hz. We exploited this in calculating spectral XCOHs between stimulus contrast-modulation and corresponding EEG. Although this approach may seem overly complicated in case of rhythmic-stimulation driven SSRs, it was mandatory for studying stimulus-locked continuous brain responses to frequency-varying stimulation in the other conditions of our experiment (see *Step 3*). Thus applying it to SSRs and comparing it to estimates of SSR power and ITC provided a proof-of-principle for subsequent analyses. Put differently, our approach required a demonstration that our measure of SSR stimulus locking was as sensitive to top-down attentional biases as were SSR power and ITC.

Because analyses of stimulus locking to rhythmic and quasi-rhythmic visual stimulation were highly similar they are described in detail below (see section *EEG-stimulus locking to quasi-rhythmic band-limited stimulation*). Most importantly, this analyses yielded spectral representations of phase XCOH between stimulus and EEG. Systematic attentional modulation of XCOH was assessed similarly to SSR power and ITC and aggregated into AMIs (see Eq. (2)).

All AMIs were subjected to a two-way repeated-measures ANOVA with factors of *SSR measure* (power, ITC and XCOH) and *stimulus position* (10 Hz, left vs 12 Hz, right). We further tested whether AMIs based on the established SSR measures power and ITC predicted attentional modulation in XCOH. Because attentional modulation was comparable between left and right stimuli (see *Results*), we performed regression analyses (i.e. linear model fits) on AMIs collapsed across stimuli. Two separate regressions tested for linear dependencies of XCOH gain effects on SSR power and on ITC attentional modulation.

#### 2.6.4 Step 3: EEG-stimulus locking to quasi-rhythmic band-limited stimulation

As the key element in determining whether the EEG phase-locked to frequency-varying visual stimulation, we calculated the spectral cross-coherence (XCOH) between EEG time series and corresponding contrast modulation functions (CMFs, Fig. 1b). To this end, artefact-free epoched EEG time series of all conditions were down-sampled (using the Fieldtrip function *ft_resampledata*) to the sampling rate of CMFs (100 Hz, i.e. the screen refresh rate). A built-in low-pass filter, applied before down-sampling, served to avoid aliasing artefacts. Resampled epochs were truncated to 3-s segments starting 0.5 s after stimulus onset. This step removed edge effects introduced by the low-pass filter ('filter ringing'; Widmann et al., 2015) and excluded strong transient brain responses evoked by stimulus onset. Down-sampled EEG scalp voltage time series were converted to scalp current densities.

Subsequently, data segments as well as corresponding CMFs of each trial were re-epoched into five successive 1-s segments with an overlap of 0.5 s and then subjected to Fourier transforms using the multi-taper method as implemented in Fieldtrip (Percival and Walden, 1993) with a spectral smoothing constant of ±2 Hz. As an exception, in XCOH analyses of constant-stimulation conditions single Hanning tapers were applied to the data. 1-s data segments (=100 sampling points) were zero-padded to a length of 2 s prior to Fourier transforms to allow for a frequency resolution of 0.5 Hz. Using thus obtained complex Fourier spectra we calculated the XCOH of each EEG sensor with each of the two CMFs separately (by means of Fieldtrip’s *ft_connectivityanalysis*, method ‘coh’). We pooled data across both attention conditions (*Attend Left* vs Attend *Right*) to substantiate a general EEG-stimulus locking. Following above described steps thus yielded XCOH spectra for both stimuli and for each of the three frequency-varying stimulation conditions.

Additionally, we calculated a surrogate XCOH using time-reversed contrast modulations (Gross et al., 2013, Park et al., 2016) to assess whether resulting peaks in XCOH were a by-product of stimulating within respective frequency bands or indeed reflected the stimulus contrast modulations on each trial precisely. XCOH spectra based on original vs reversed contrast modulations were then compared by means of cluster-based permutation tests.

As documented in the *Results* section below, we found substantial EEG-stimulus locking to both stimuli regardless of their frequency band, which afforded further comparisons of peak XCOH between conditions. For that purpose we averaged XCOH (calculated based on original contrast modulations) across frequencies and electrodes separately for each condition and for left and right stimuli. To control for differences in cluster sizes (number of electrodes) and frequency bandwidth between conditions we considered 11 recording sites showing maximum coherence in each condition (=minimum cluster size, beta band stimulation) and ±3 spectral components, i.e. ±1.5 Hz, around respective centre frequencies (=minimum bandwidth, theta band stimulation). A repeated-measures ANOVA with factors of *stimulus frequency* (3 factor levels: theta, alpha, beta) and *stimulus position* (2 factor levels: left vs right) subsequently evaluated differences in EEG-stimulus locking.

#### 2.6.5 Step 4: modulation of EEG-stimulus locking by attention

We repeated above described processing steps while keeping data of conditions *attend left* and *attend right* separate to evaluate whether the allocation of spatial attention towards a stimulus modulated corresponding EEG-stimulus locking. For each stimulus we thus obtained one XCOH spectrum under the condition that the stimulus was attended and a second one under the condition that the stimulus was unattended. Again, both spectra were also derived using time-reversed stimulus functions. These surrogate spectra were subtracted from the original XCOH spectra to retain only the amount of XCOH (=corrected XCOH) that could be attributed to precise EEG-stimulus locking.

Group level systematic gain was assessed by means of cluster-based permutation testing corrected XCOH at the centre frequencies of respective stimulated frequency bands. Note that only channels exhibiting significant EEG-stimulus locking (see *step 3*) were regarded in this analysis (i.e. were considered in setting up the channel neighbourhood structure for the cluster-based testing procedure). Because only few channels showed substantial locking to beta-band stimulation this condition was excluded from analyses of gain effects.

To increase statistical sensitivity towards the expected positive gain effects we employed one-tailed testing. Substantial gain effects during theta and alpha-band stimulation (see Results) were compared by means of a two-way repeated-measures ANOVA with factors of *stimulus frequency* (theta vs alpha) and *stimulus position* (left vs right). Finally, two separate linear-model fits explored dependencies of XCOH attentional modulation during theta- and alpha-band stimulation with XCOH gain effects during strictly rhythmic stimulation (see *step 2*).

## 3 Results

### 3.1 Behavioural data

**Fig. 3:**
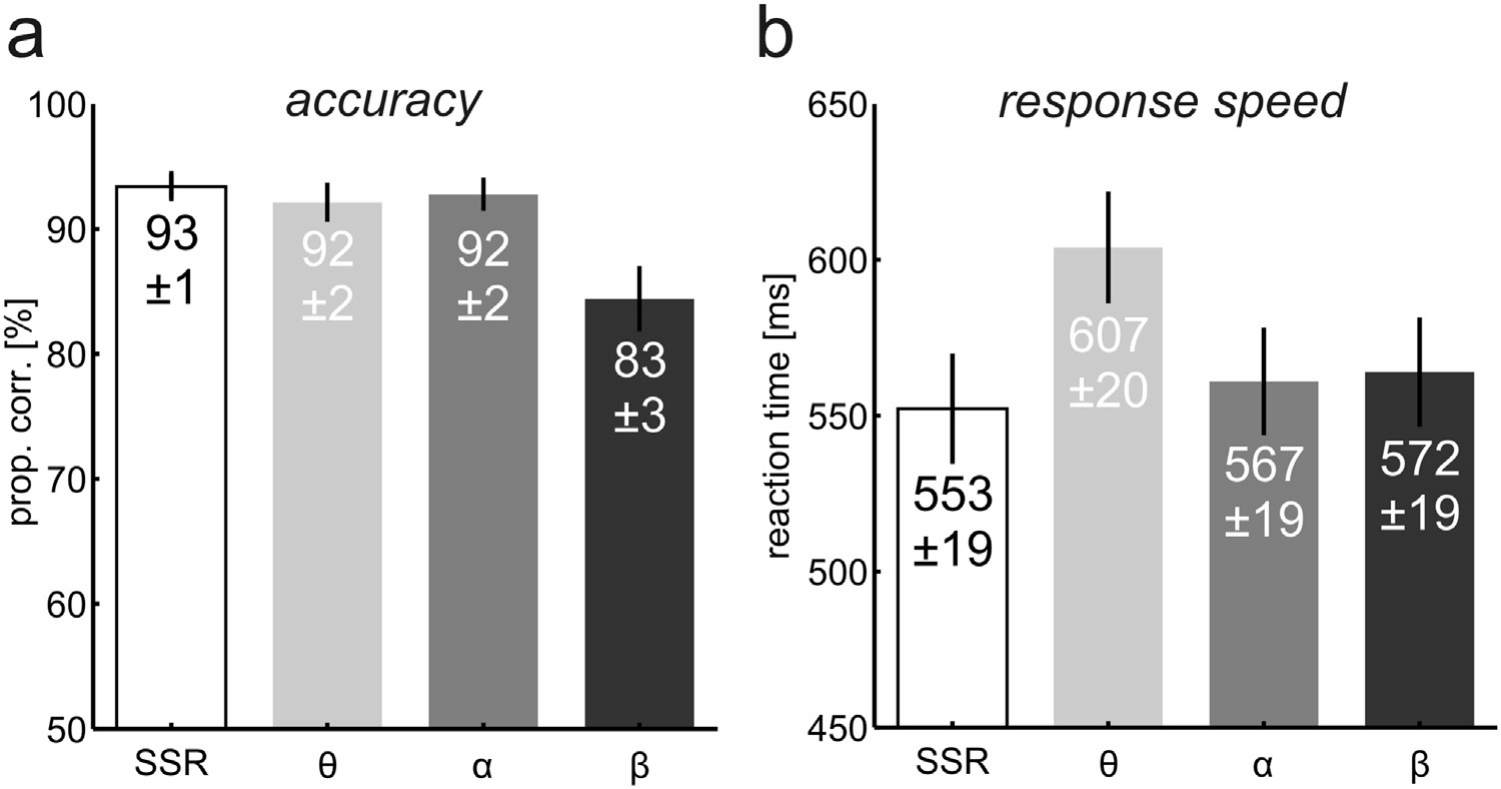
Results of behavioural performance analyses, collapsed across left and right visual stimuli. (a) Accuracy as expressed in proportion correct in %. (b) Response speed (median RT) in msec. Error bars in (a) and (b) indicate standard errors of the mean (*SEM*). Values on bars display Mean±*SEM.* S*SR=*stimulation with constant frequencies (10|12 Hz), θ=theta band (4–7 Hz), α=alpha band (8–13 Hz), and β=beta band stimulation (14–20 Hz).

Participants responded with similar accuracy to transient “flashes” while attending to left vs. right patches (main effect *stimulus position*: *F*(1,16)<1). Accuracy however depended on the rate of stimulus contrast modulation (main effect *stimulus frequency*: *F*(3,48=12.05, p_GG_<0.001, ε_GG_=0.54, ƞ^2^=0.19) and was lowest when Gabor patches where flickering in the beta range (Fig. 3*a*). The interaction of both factors was insignificant (*F*(3,48)=1.13, p=0.35).

Response speed analyses also revealed an influence of stimulus frequency (*F*(3,54)=13.18, p_GG_<0.001, ε_GG_=0.66, ƞ^2^=0.06). Relatively slow responses during theta band stimulation likely caused this effect (Fig. 3b). The *stimulus position* (*F*(1,16)=1.13, p=0.31) and an interaction of both factors had negligible effects on reaction times (*F*(3,48)<1).

Participants produced on average 0.24 false alarms (*SEM*±0.15) per condition with an individual maximum of 3 false alarms in one condition. False alarms were not further analysed due to their overall low occurrence.

Taken together, these data indicate that participants were well able to maintain attentional focus on the cued stimulus.

### 3.2 Electrophysiological data

#### 3.2.1 Different SSR measures reveal comparable attentional gain

**Fig. 4:**
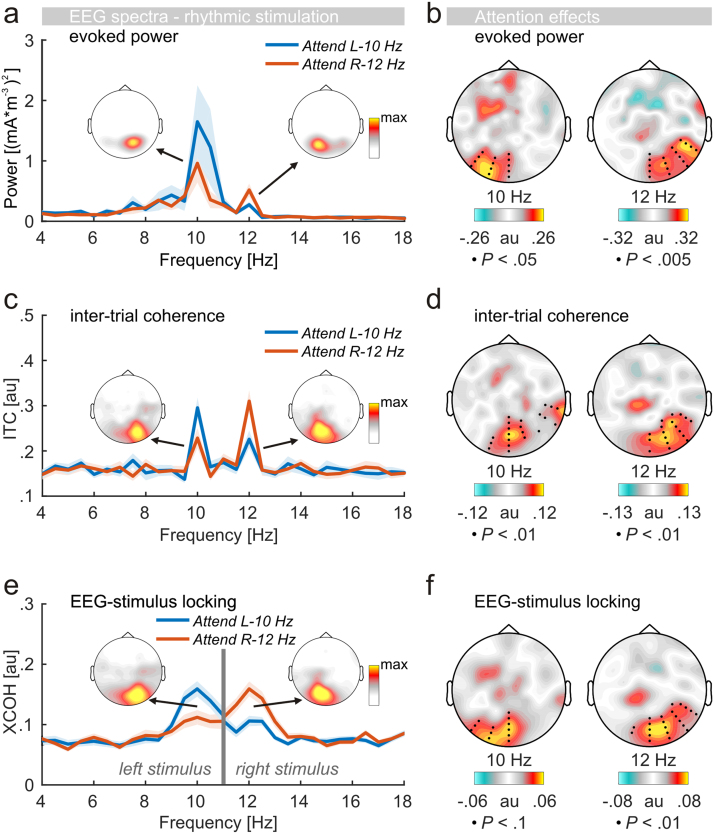
SSRs driven by rhythmic stimulation – spectra and attention effects. (a) Grand average EEG scalp current density power spectra based on Fourier transforms of averaged epochs (thus ‘evoked’) for *Attend Left* (Blue line) and *Attend Right* conditions (Red line). Peaks at 10 and 12 Hz correspond to the stimulation frequencies. Shaded areas depict standard error of the mean (*SEM*). Inset scalp maps illustrate topographical distributions of power and highlight the lateralization of respective maxima. (b) Scalp maps depicting power differences (*Attended* minus *Unattended*). Black dots indicate electrode clusters that showed systematic modulations as confirmed by cluster-based permutation statistics. The corresponding *P*-Value is given below each map. (c,d) Same as in (a) and (b) but for SSR inter-trial coherence (ITC). (e) Same as in (a) but for cross-coherence (XCOH; i.e. EEG-stimulus locking). Note that this analysis yields two sets of spectra – one set of two (*Attend Left* and *Attend Right*) for the XCOH with each stimulus. For illustrative purposes the plot is split halfway such that the left part, up to 11 Hz, shows spectral XCOH with the left stimulus (10 Hz) and the right part, from 11 Hz on, shows XCOH with the right stimulus (12 Hz). (f) Same as in (b) but for XCOH.

Condition-resolved spectra showed greater SSR power, greater ITC and greater EEG-stimulus locking (XCOH) at Fourier components corresponding to the stimulation frequencies (10 and 12 Hz) when either stimulus was attended (Fig. 4a, c and e). Cluster-based permutation tests based on scalp topographies (Fig. 4b, d and f) confirmed these effects statistically (all *P*<.05) for both stimuli and all three SSR measures (power, ITC vs XCOH). In case of the 10 Hz stimulus XCOH, the attention effect was only marginally significant (*P<*.1).

**Fig. 5:**
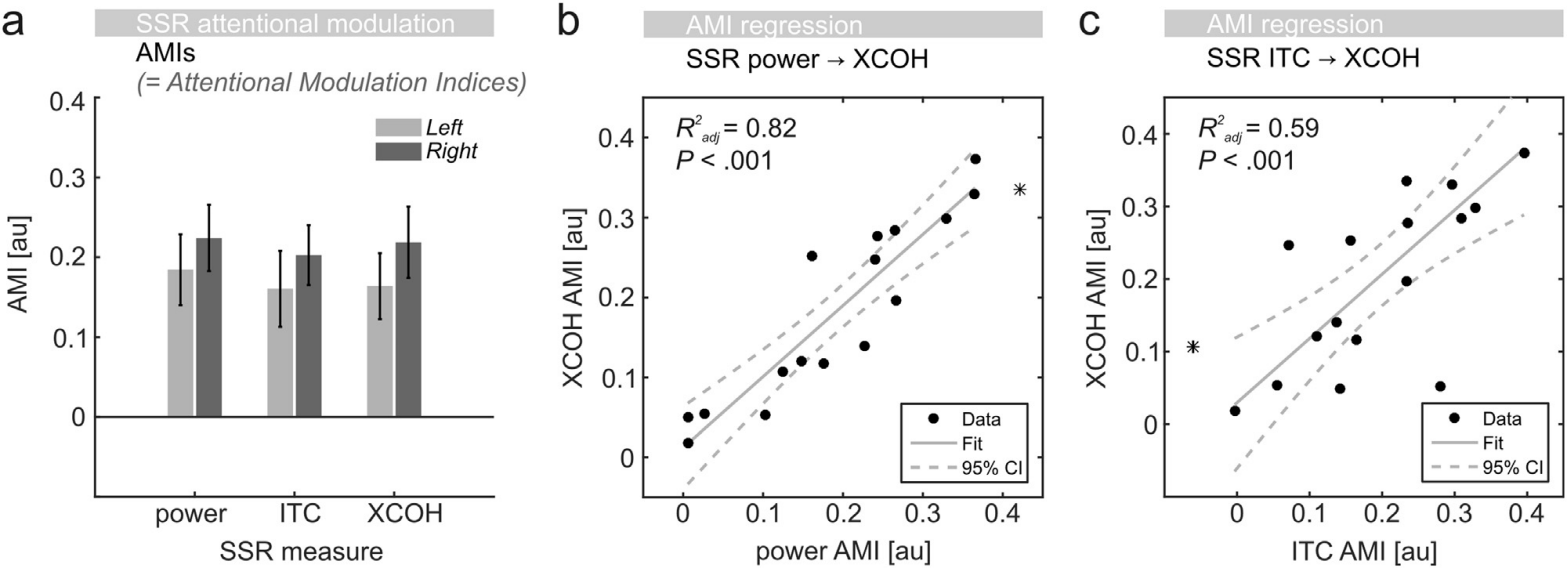
Attentional modulation of brain responses (SSRs) driven by rhythmic visual stimulation. (a) Attentional modulation indices (AMIs) for left (light grey) and right (dark grey) stimuli based on SSR power, inter-trial phase coherence (ITC) and EEG – stimulus locking (XCOH). (b) SSR power gain effects (x-axis) predict individual attentional modulation of XCOH. Black dots represent participants. Straight grey line=robust linear fit. Dashed lines=95%-confidence intervals (CIs). Adjusted R^2^ values (upper left corner) display goodness-of -fit. (c) Same as in b but using ITC-based AMIs as predictor (x-axis). Asterisks in b and c indicate outliers, identified by means of Cook’s distance, not included in the regression.

To quantify attention effects, we computed attention modulation indices (AMIs) according to formula (2) based on SSR power, ITC and XCOH, collapsed across respective significant electrode clusters each (Fig. 5a). Specific contrasts against zero showed that AMIs indicated substantial gain in all six cases (all *P*<.05, Bonferroni-corrected for multiple comparisons).

A repeated-measures ANOVA established that AMIs were comparable across the three SSR *measures* (*F*(2,32)<1) and for left and right *stimulus positions* (*F*(1,16)<1) although average AMIs suggested a slight advantage for the SSRs driven by the right stimulus (Fig. 5a). The interaction *stimulus positions*×*measures* remained negligible (*F*(2,32)<1). Fitting robust linear models (see Fig. 5b and c) further demonstrated that individual gain effects in SSR stimulus locking (XCOH) were predicted by SSR amplitude gain (one outlier removed; *F*(1,14)=67.00, *P*<0.001, adjusted *R*^*2*^=0.82), as well as ITC gain (one outlier removed; *F*(1,14)=22.20, *P*<0.001, adjusted *R*^*2*^=0.59).

#### 3.2.2 Quasi-rhythmic contrast modulation gives rise to EEG-stimulus locking

**Fig. 6:**
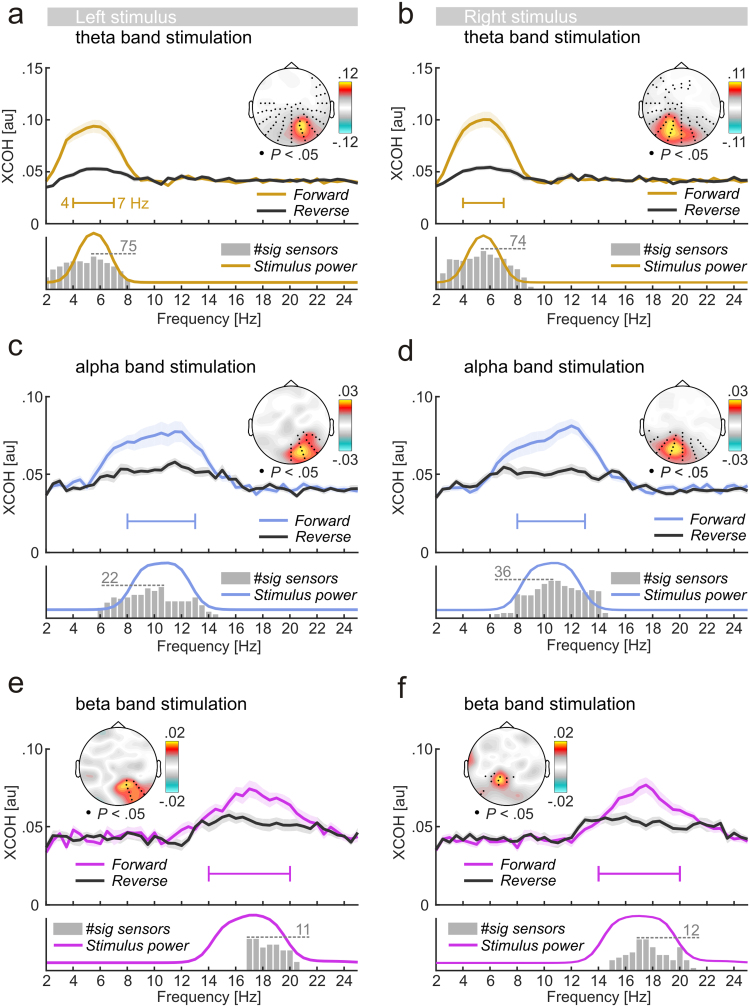
EEG-stimulus locking and EEG power spectra during stimulation. (a) Main plot: Grand average cross coherence (XCOH) spectra calculated based on original forward (coloured line) and reversed stimulus functions (black line) of the left stimulus flickering with theta-band frequencies. Shaded areas indicate standard errors of the mean. Inset scalp map: Topographical distribution of the difference between *Forward* and *Reverse* XCOH. Black dots depict the cluster of electrodes that exhibited systematic stimulus locking at the centre frequency of the stimulation bandwidth (5.5 Hz for theta band stimulation). Side plot (below main plot): The coloured line shows the average spectral distribution of stimulus power on an arbitrary scale. Grey bars illustrate significant cluster sizes at each frequency (dashed line indicates max size). (b,c,d,e,f) Same as in (a) but for different frequency bands (left column) and for stimuli presented to the right (right column).

The present study mainly aimed at investigating how the visual system responded to quasi-rhythmic stimulation. We found that these brain responses were characterised by an EEG stimulus-locking restricted to frequency bands featured in the stimulation. More specifically, XCOH spectra quantifying EEG-stimulus locking in Fig. 6a-f showed clear peaks during theta (4–7 Hz), alpha (8–13 Hz) and beta band stimulation (14–20 Hz). When tested against surrogate data based on EEG-stimulus locking with time-reversed stimuli, frequency ranges exhibiting substantial EEG-stimulus locking (all<EEG channel×frequency>clusters: *P*<.05) remarkably resembled spectral profiles of corresponding stimuli (Fig. 6a-f, compare XCOH spectra with corresponding stimulus power spectra beneath).

While these results highlighted the ability of the visual system to follow stimulus-specific frequency changes in time, topographical distributions (scalp maps) of XCOH in Fig. 6 suggested that responses to both stimuli could further be separated in space: Peak cross coherence was lateralized to the hemisphere contralateral to the location of the corresponding stimulus for each frequency band.

A comparison of peak XCOH between stimulation conditions (*theta:* 0.146±0.015, *alpha:* 0.085±0.008, *beta:* 0.072±0.004; all *Mean*±*SEM*, collapsed across left and right stimuli) confirmed the monotonous drop in EEG-stimulus locking from low to high frequency bands (main effect *stimulus frequency*: F(2,32)=31.03, p_GG_<0.001, ε_GG_=0.67, η^2^=0.29; also see Fig. 6a-f). XCOH remained comparable between left and right stimuli (main effect *stimulus position*: F(1,16)<1). An interaction of both factors was not significant (F(2,32)<1).

**Fig. 7:**
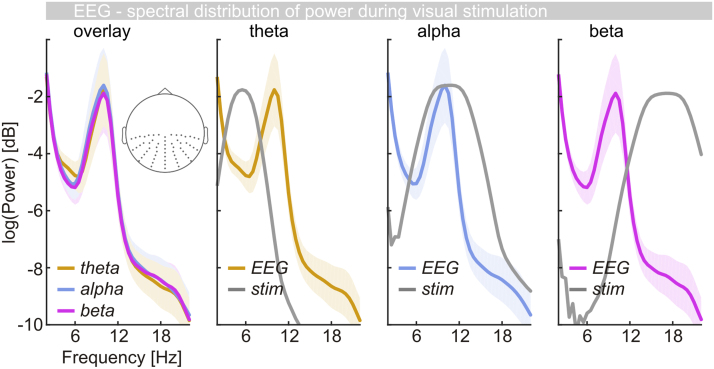
EEG power spectra during stimulation. Leftmost panel: Overlay of EEG power (logarithmic scale) during theta- (yellow), alpha- (blue) and beta band (purple) visual stimulation. Spectra were pooled across electrodes indicated on the inset scalp map. Other panels: Individual spectra from (a) superposed with the spectral composition of visual stimuli (grey line) in respective conditions. Note that stimulus power is arbitrarily scaled – peak stimulus power was set to peak alpha power for illustrative purposes.

Interestingly, when only considering EEG spectral power during stimulation (Fig. 7) there was no indication of stimulus-related frequency-specific neural activity. In fact, power spectra – obtained from the same spectral decomposition steps as XCOH spectra – were virtually identical irrespective of the stimulated frequency band. Consistently, they showed the typical 1/f characteristic and a prominent alpha peak.

#### 3.2.3 Attention modulates EEG-stimulus locking in theta- and alpha-bands

**Fig. 8:**
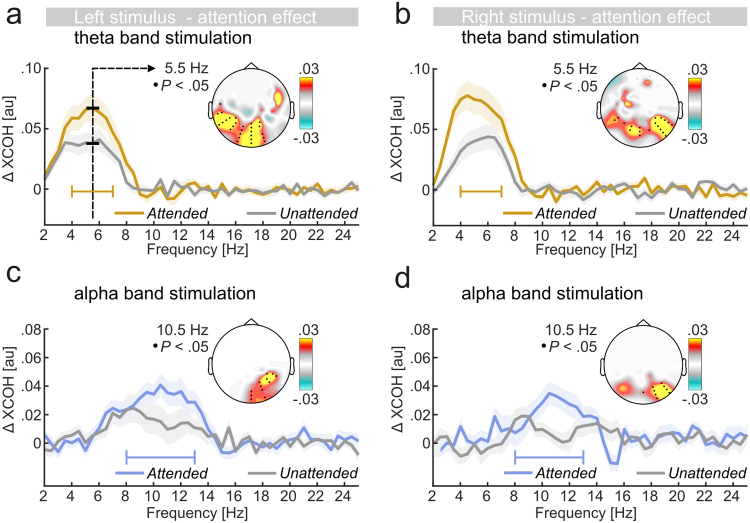
EEG-stimulus locking: modulation by attention. (a-d) Grand-average spectra show greater cross-coherence (XCOH) when left and right stimuli were attended (coloured line) vs unattended (grey line). Shaded areas represent standard errors of the mean. Inset scalp maps depict the topographical distribution of the difference between *Attended* and *Unattended* XCOH. Black dots in scalp maps indicate clusters of electrodes that exhibited systematic gain effects at the centre frequencies of respective stimulation bandwidths (as illustrated in a).

**Fig. 9:**
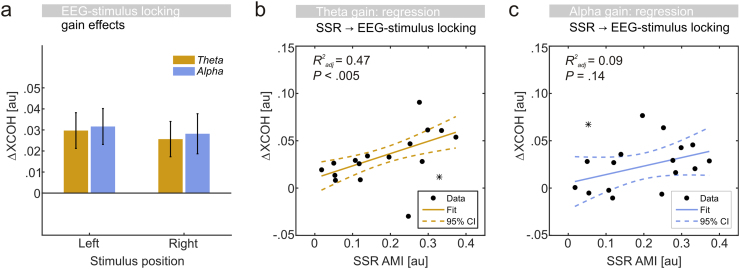
Gain modulation of EEG-stimulus locking – comparisons: (a) Gain effects, expressed as differences in XCOH (attended minus unattended) for theta- (orange) and alpha-band (blue) EEG-stimulus locking driven by left and right stimuli. (b) Modulation of SSR stimulus locking (x-axis) predicts individual modulation of theta-band stimulus locking (y-axis). Black dots represent individual participants. Straight line=robust linear fit. Dashed lines=95%-confidence intervals (CIs). Adjusted R^2^ values (upper left corner) display goodness-of -fit. (c) Same as in b but regressing gain modulation of alpha-band stimulus locking (y-axis). Asterisks in b and c indicate outliers, identified by means of Cook’s distance, not included in the regression.

Scalp maps in Fig. 8 depict electrode clusters (as determined by cluster-based permutation tests) that showed systematic gain effects (all *P*<.05, one-sided) in theta- and alpha-band stimulation conditions. Gain effects were further readily observable within stimulated frequency bands in XCOH spectra (Fig. 8a-d). A comparison (repeated measures ANOVA) of gain effects pooled across electrodes of respective clusters (Fig. 9a) showed that attentional modulation neither varied with the *stimulus frequency* band (*F*(1,16)<1) nor with the *stimulus position* (*F*(1,16)<1). No systematic interaction between factors was observed (*F*(1,16)<1).

Comparable gain effects for left and right stimuli afforded collapsing across the factor *stimulus position* in further regression analyses. Here, individual gain effects on SSR stimulus locking (depicted in Fig. 4e and f) were found to predict gain effects on theta-band (one outlier excluded, *F*(1,14)=14.00, *P*<0.005, adjusted *R*^*2*^=0.47) but not on alpha-band EEG-stimulus locking (one outlier excluded, *F*(1,14)=2.64, *P*=0.14, adjusted *R*^*2*^=0.09) although the latter followed a similar trend (Fig. 9b-c).

## 4 Discussion

We studied how mass neural activity reflects quasi-rhythmic sensory input. Our data demonstrate that the visual system faithfully follows dynamics of stimuli changing contrast on functionally relevant and ecologically plausible time scales. Corresponding neural activity was characterized by a sustained phase-locking between EEG and stimulation whereby higher frequencies led to lower coupling between EEG and stimulus. For theta- and alpha-band stimulation EEG-stimulus locking increased when participants attended to the location of corresponding stimuli. For theta-band stimulation attentional modulation closely resembled individual gain effects on steady-state responses (SSRs) driven by strictly rhythmic stimulation.

### 4.1 Tracing the temporal structure of visual input

The dynamics of our visual environment endow continuous sensory input with rich temporal structure (Blake and Lee, 2005) – a feature upon which Human visual perception heavily relies when segmenting scenes into objects or when extrapolating stimulus trajectories. Here, we demonstrate that neural activity continuously reflects stimulus temporal structure.

Strikingly, related findings have been reported from animal single-cell recordings: For instance, Buracas et al. (1998) demonstrated that spike rates of extra-striate visual neurons in alert primates encoded the fine temporal structure of drifting Gabor patches that changed movement direction stochastically between preferred and anti-preferred directions. The authors further found that these time-varying stimuli were easier discriminable than Gabor patches constantly drifting in one direction when their stochastic temporal structure was defined on an ecologically relevant time scale (30–300 ms). This scale corresponds well with constants in saccade behaviour (Buschman and Miller, 2009, Otero-Millan et al., 2008) and approximately marks the minimum (250 ms at 4 Hz) and maximum periods (50 ms at 20 Hz) of the here employed stimulation. Such a stimulus-locked modulation of neuronal spike rate has since been described in different species and using a variety of stimuli (Bair and Koch, 1996, Berry et al., 1997, Butts et al., 2007, de Ruyter van Steveninck et al., 1997).

More recently, the relative phase of low-frequency oscillating (i.e. <20 Hz) local field potentials (LFPs) in the animal brain has been shown to influence spike rates and thus contributes additional information about dynamic visual scenes (Mazzoni et al., 2011, Montemurro et al., 2008). Montemurro et al. (2008) further reported a monotonous relationship in LFP oscillations below 20 Hz: the lower their frequency the higher the amount of information they code for. Their finding provides a possible explanation for the preponderance of the visual system to trace low-frequency visual input as reflected in our finding of decreasing EEG-stimulus-locking with increasing stimulation frequency range. As laid out in detail below, these results interface with the idea that LFPs can phase-lock, or *entrain*, to dynamic continuous sensory stimulation.

### 4.2 EEG-stimulus locking and entrainment of intrinsic oscillations

Low-frequency (<20 Hz) brain oscillations have been ascribed a vital role in parsing sensory input (Ghitza, 2012, Schroeder and Lakatos, 2009, VanRullen et al., 2014). The notion of entrainment assumes that these ongoing oscillations can re-align their phases to temporal regularities in sensory input as to facilitate this sampling process (Lakatos et al., 2008). Although the majority of studies on visual entrainment feature strictly rhythmic stimulation, Calderone et al. (2014) recently pointed out that some have also looked into quasi-rhythmic scenarios thus extending the notion of entrainment to more naturalistic stimuli. For example, Besle et al. (2011) demonstrated cortical entrainment to an approximate delta-rhythmic stimulation (mean frequency 1.5 Hz) in Human electrocorticographic (ECog) recordings. However, comparing strictly-rhythmic and quasi-rhythmic stimulation in the same frequency range, Cravo et al. (2013) reported stronger entrainment to the former.

Another line of recent research employed strictly rhythmic visual stimulation in the ~10 Hz range while considering resulting periodic modulations in neural activity as entrainment of intrinsic generators of the parieto-occipital alpha rhythm (de Graaf et al., 2013, Mathewson et al., 2012, Notbohm et al., 2016, Spaak et al., 2014). Some of these studies used quasi-rhythmic (Mathewson et al., 2012) or irregular^1^ visual stimulus sequences (Notbohm et al., 2016, Spaak et al., 2014) as control conditions because intrinsic oscillators should resonate less (or not at all) with frequency-varying sensory input. Analyses of neural responses in respective conditions, if carried out, were indeed unable to substantiate entrainment (Mathewson et al., 2012, Notbohm et al., 2016). Specifically, Mathewson et al. (2012) found that EEG phase locking in the stimulated frequency range during quasi-rhythmic (“variable”) conditions was indistinguishable from another condition with no intermittent stimulation. Taken together, studies into entrainment to frequency-varying sensory input have so far reported equivocal findings and support only relatively weak entrainment to low-frequency (delta-band) quasi-rhythmic stimulation.

Conflicting findings likely relate to a methodological issue: Variable-frequency brain responses are more difficult to analyse by means of standard spectral decomposition because their frequency-domain representations distribute across the spectrum. This yields signal-to-noise ratios inferior to constant frequency responses such as SSRs that concentrate their spectral power in a single frequency component (Norcia et al., 2015). Variations in frequency violate the stationarity assumption of widely applied Fourier-transform based approaches. Put differently, when applying a Fourier transform to a signal one assumes that the oscillations composing the signal are constant in frequency over time (Cohen, 2014, Gross, 2014) – a requirement that is hardly ever met by noisy EEG time series of several seconds duration let alone the natural sensory input that shapes them.

Studies into cortical processing of audio-visual speech, a prime example for quasi-rhythmic stimulation (Chandrasekaran et al., 2009), have circumvented this limitation to some extent by taking into account the time-varying nature of the stimulus signal (Crosse et al., 2015, Gross et al., 2013, Zion Golumbic et al., 2013). For example, Gross et al. (2013) directly computed the spectral dependencies between time- and frequency varying stimuli and brain signals (also see Peelle et al., 2013). Applying a related approach, the present results support the observation that the visual system traces quasi-rhythmic stimulation in the speech-relevant theta frequency range (4–7 Hz, Park et al., 2016). Our finding of most pronounced EEG locking to stimulation fluctuating within the theta-band may highlight a special role for narrow-band low frequency periodic brain responses. Nevertheless, substantial EEG stimulus-locking to higher and broader frequency ranges (here: alpha and beta) suggests a more general tracing process that codes the temporal structure of continuous input in visual cortex. Beyond facilitating visual processing of speech, tracing the dynamics of visual stimuli on different time scales may subserve multisensory integration with temporally synchronized sensory input to other senses (Parise and Ernst, 2016, Talsma et al., 2010, van Atteveldt et al., 2014) as has been demonstrated with quasi-rhythmic visual input (Schall et al., 2009, Van der Burg et al., 2008).

It remains to be seen, however, whether such a tracing process can be fully accounted for by assuming entrainment of ongoing brain oscillation (Besle et al., 2011, Mathewson et al., 2012, Spaak et al., 2014). Alternatively, contributions from stimulus-evoked activity may have to be considered (Capilla et al., 2011, Keitel et al., 2014, Thut et al., 2011). In our data this issue is most evident in a strong alpha band response that dominates the EEG power spectrum in all conditions. This signature of intrinsic rhythmic activity (Keitel and Gross, 2016) seems to remain unaffected in power and peak frequency regardless of stimulated frequency ranges (Fig. 7) and will be investigated in a separate dedicated analysis of the present data.

### 4.3 Multiple frequency-varying stimuli allow tracking attentional allocation

Speech entrainment studies established that attending to a specific source in a multi-speaker scenario enhances tracking precision of the speech signal, i.e. preferential entrainment to the attended input (Rimmele et al., 2015, Zion Golumbic et al., 2013, Zoefel and VanRullen, 2015) relative to irrelevant concurrent input. The present data explicitly demonstrate that this effect generalizes to situations in which observers attend to one of two concurrent but spatially separated visual inputs with individual temporal structure within theta or alpha frequency ranges. Thus, the structure of continuous visual input at an attended position may be traced more precisely by enhancing neural phase-locking to stimulation at that position (Chennu et al., 2009).

Our measure of EEG-stimulus locking allowed separating neural responses to simultaneously presented stimuli similar to steady-state responses (SSRs) to strictly rhythmic stimulation. Employing frequency-varying stimulation can thus be regarded as an extension of the frequency-tagging approach (Norcia et al., 2015) that alleviates the necessity of tagging multiple stimuli with distinct but steady frequencies. Instead, multiple stimuli can be tagged with frequencies that vary within a common band rendering them perceptually similar. Further paralleling SSRs, theta- and alpha-band EEG-stimulus locking increased with allocation of spatial attention to the position of the driving stimulus, an effect that allows tracking the attentional focus. Still, low beta-band EEG-stimulus locking points at practical limitations of the frequency-varying approach. Also, when comparing scalp maps of attention effects between SSR-stimulus locking (Fig. 4f) and EEG-stimulus locking to theta- and alpha band stimulation (Fig. 8a-d), the latter seems to be more far-spread, which could be due to frequency-varying stimulation involving additional or different cortical generators (Keil et al., 2009b, Muller et al., 1997). Additional studies will need to determine critical parameters (frequency band, bandwidth) for attentional modulation.

Lastly, note that attention effects on SSRs during strictly-rhythmic (i.e. constant flicker) conditions consistently but counter-intuitively located contra-laterally to the respective power, ITC and XCOH maxima (compare topographies between left and right columns of Fig. 4). Frequency-tagging experiments investigating effects of spatial attention on lateralized stimuli have reported similar topographical effects (Keitel et al., 2013a). Expecting an attention effect contra-laterally to the attended side in topographical contrasts entails the implicit assumption that attention effects exclusively manifest as stationary response gains. Alternatively, however, attention to a stimulus may lead to the recruitment of additional neuronal ensembles generating SSRs – for example accessing extended networks in upstream visual areas (Lithari et al., 2016, Zhang et al., 2015). Instead of increasing a local maximum this would result in more far-spread topographies of responses driven by attended stimuli. Consequentially, seemingly ipsilateral attention effects could be produced by contrasting far-spread (attended) with more circumscribed (unattended) maxima in topographies. SSR sensor space analysis typically circumvents this issue by analysing data collapsed across electrode clusters. Recent advances in deriving spatial filters for SSR analyses may provide more insight and a possible remedy (Cohen and Gulbinaite, 2016).

### 4.4 Fluctuating rhythms: in-between strictly periodic and aperiodic

In this study we mainly focussed on some characteristics of brain responses to quasi-rhythmic stimulation and comparing these with SSRs to strict rhythms. When considering rhythmicity as a continuous physical property of sensory input signals, however, our quasi-rhythmic case can still be considered close-to-rhythmic in comparison to another long-standing line of research employing arrhythmic, spectrally broadband stimulation that mainly aims to characterise response properties (e.g. impulse response) of the visual system (Sutter, 2001). In one such study, for example, VanRullen and Macdonald (2012) measured responses to broadband (1–80 Hz) luminance fluctuations and reported evidence for an alpha-reverbatory process that indicated a prolonged (~1 s) re-iteration of perceptual episodes after exposure – a finding impossible to obtain using (quasi-) rhythmic stimulation. Other studies employed so-called m-sequences (Bin et al., 2011, Sutter, 1992) or a particular derivative, Gold-codes (Thielen et al., 2015), primarily as a means to increase performances of brain-computer interfaces. Brain responses to these pseudo-random binary sequences (e.g. contrast reversals) can be quantified by cross-correlating stimulus- and EEG/MEG-recorded brain signals, an approach that is related to the here employed spectral cross-coherence. Thus obtained measures of brain-stimulus correspondence have been shown to indicate attentional allocation in multi-element displays (Slotnick et al., 2002) similar to SSRs and the present measure of EEG-stimulus locking.

In sum, brain responses to arrhythmic stimulation have been studied extensively and it stands to question whether extrapolating respective findings may trivialize the present results. However, arrhythmic stimulation has in many ways been optimised to characterise the basic physical properties of the visual system as an “input filter”. Our quasi-rhythmic stimuli instead comprise perceptual experiences that are physically plausible in terms of their spectro-temporal composition – as is evident when comparing them to of speech (Chandrasekaran et al., 2009). Moreover, it is possible that although stimulus rhythmicity can be conceived of as a quantitative continuum (from strictly rhythmic to arrhythmic, or, from a spectral perspective, single frequency to broadband), plausible quasi-rhythmic stimuli that approach ecological validity are also perceived as qualitatively different from arrhythmic stimulation and therefore warrant dedicated studies.

### 4.5 Conclusion

We found that EEG-recorded brain responses continuously reflect quasi-rhythmic dynamics in visual stimulation across different time scales. Moreover, multiple simultaneously presented stimuli that displayed independent dynamics were traced individually – arguably a favourable skill when navigating and behaving adaptively in an ever-changing visual environment. Supporting this notion, our measure of brain-stimulus coupling increased (for theta- and alpha-band stimulation) when corresponding stimuli were behaviourally relevant. These gain effects possibly signify that the visual system traces attended dynamic stimulation with enhanced temporal precision.

## Conflict of interest

The authors declare no competing financial interests.

## Footnotes

1 Note that due to the construction of irregular stimuli in respective studies, these are spectrally non-uniform with broader peaks around similar frequencies as in regular conditions (see e.g. Notbohm et al., 2016, Fig. 1). Thus, they retain some periodicity and can also be considered quasi-rhythmic in the present sense.
